# Incidence and Risk of Treatment-Related Mortality with Anti-Epidermal Growth Factor Receptor Monoclonal Antibody in Cancer Patients: A Meta-Analysis of 21 Randomized Controlled Trials 

**DOI:** 10.1371/journal.pone.0081897

**Published:** 2013-11-28

**Authors:** Xing Li, Bao-En Shan, Juan Wang, Lian-Ping Xing, Xiao-Jin Guo, Yue-Hua Zhang, Peng-Hui Shi, Zhi-Yu Wang

**Affiliations:** 1 Department of Biotherapy, Fourth Hospital of Hebei Medical University, Shijiazhuang, People’s Republic of China; 2 Centre of Scientific Research, Fourth Hospital of Hebei Medical University, Shijiazhuang, People’s Republic of China; 3 Department of Pathology and Laboratory Medicine, University of Rochester Medical Center, Rochester, New York, United States of America; Rush University, United States of America

## Abstract

**Background:**

Anti-epidermal growth factor receptor (EGFR) monoclonal antibodies (MoAbs) cetuximab and panitumumab have emerged as an effective targeted therapy in the treatment of cancer patients, but the overall incidence and risk of fatal adverse events (FAEs) associated with these agents is still unclear.

**Methods:**

Databases from PubMed, Web of Science and abstracts presented at ASCO meeting up to May 31, 2013 were searched to identify relevant studies. Eligible studies included prospective randomized controlled trials evaluating MoAbs in cancer patients with adequate data on FAEs. Statistical analyses were conducted to calculate the summary incidence, odds ratio and 95% confidence intervals (*CI*s) by using either random effects or fixed effect models according to the heterogeneity of included studies.

**Results:**

A total of 14,776 patients with a variety of solid tumors from 21 clinical trials were included in our analysis. The overall incidence of MoAbs associated FAEs was 1.7% (95%CI: 1.1–2.5%), and the incidence of cetuximab-related FAEs was higher than that of panitumumab (2.0% versus 0.9%). Compared with the controls, the use of MoAbs was associated with a significantly increased risk of FAEs, with an OR of 1.37 (95%CI: 1.04–1.81, *p*=0.024). Subgroup analysis based on EGFR-MoAbs drugs, phase of trials and tumor types demonstrated a tendency to increase the risk of FAEs, but the risk did not increase in breast cancer, esophagus cancer and phase II trials.

**Conclusions:**

With present evidence, the use of EGFR-MoAbs is associated with an increased risk of FAEs in patients with advanced solid tumors.

## Introduction

Epidermal growth factor receptors (EGFR) are a large family of receptor tyrosine kinases which are overexpressed in many types of cancer[1], including breast[2], lung[3,4], esophageal[5], and head and neck[6]. EGFR and its family members are the major contributors of a complex signaling cascade that modulates proliferation, anti-apoptosis, differentiation, adhesion, migration and survival of cancer cells[7]. Due to their multidimensional role in the progression of cancer, EGFR and its family members have emerged as attractive candidates for anticancer therapy. Currently, there are two classes of anti-EGFR agents: the monoclonal antibodies (cetuximab, panitumumab) and small-molecule tyrosine kinase inhibitors (gefitinib, erlotinib). Cetuximab (C) is a chimeric monoclonal antibody (MoAb) that binds to the EGFR and blocks the EGFR signaling cascade, thus inhibiting the growth of the tumor[8]. Panitumumab (P) is an anti-EGFR MoAb which, like C, binds to the EGFR to prevent ligand binding and inhibits the subsequent activation of key downstream signaling molecules involved in tumorigenesis[9]. Erlotinib and gefitinib are oral small molecules designed to selectively inhibit the phosphorylation of EGFR intracellular kinase domain [10,11]. In addition, these four drugs have shown clinical benefits in the treatment of many types of malignancy and have been approved for use in cancer therapy by the United States Food and Drug Administration (FDA)[10-13]. 

Fatal adverse events (FAEs) are defined as deaths that are usually secondary to the use of the pharmaceutical agent[14]. Patients with cancer may be at an increased risk because of the progressive nature of malignancy as well as the adverse events (AEs) profiles of chemotherapeutic agents. As a result, determining the incidence and risk of drugs related FAEs is important for closely monitoring and planning appropriate strategies to limit their effects. A previous meta-analysis includes a total of 13,827 patients with various advanced solid tumors from 22 phase III RCTs finds that the overall incidence of EGFR-TKIs (gefitinib and erlotinib) related FAEs is 1.9% (95%CI: 1.2-2.9%), and the use of EGFR-TKIs does not increase the risk of FAEs with an RR of 0.99 (95%CI: 0.70-1.41, *p*=0.97)[15]. However, the risk of FAEs associated with EGFR-MoAbs has not been well determined. Although a recent meta-analysis shows that cetuximab does not significantly increase the risk of FAEs in colorectal cancer (OR, 1.41; 95% CI, 0.99-2.03)[16], this meta-analysis is limited to only nine studies examining cetuximab-related FAEs in colorectal cancer, . In addition, the incidence and risk of FAEs associated with panitumumab, a newly approved anti-EGFR MoAbs, has not been assessed. Here, we conduct this meta-analysis of RCTs to determine the incidence and risk of FAEs associated with the clinical use of EGFR-MoAbs cetuximab and panitumumab. 

## Materials and Methods

### Data source

Study was conducted according to the Preferred Reporting Items for Systematic Reviews and Meta-Analyses (PRISMA) statement [17,18]. We searched the Pubmed (data from 1966 to May 2013), EMBASE (data from 1980 to May 2013), and Cochrane library databases (up to May 2013) for relevant trials. The search was conducted by using the keywords “cetuximab”, “C-255”, “Erbitux”, “panitumumab”, “Vectibix”, “randomized”, “cancer” and was limited to human studies and prospective randomized controlled clinical trials published in English. Abstracts presented at the annual meetings of the American Society of Clinical Oncology (ASCO) and the European Society of Medical Oncology (ESMO) (from 2001 to 2013) were also searched manually using the same keywords to identify relevant clinical trials; Additionally, we searched the clinical trial registration website (http://www.ClinicalTrials.gov) to obtain information on the registered randomized controlled trials (RCTs); however, only trials published in peer-reviewed publications, in full manuscript form, were included. Each publication was reviewed and in cases of duplicate publication only the most complete, recent, and updated report of the clinical trial was included in the meta-analysis

### Study selection

The goal of this study was to determine the incidence of EGFR-MoAbs associated FAEs and establish the association between treatment with EGFR-MoAbs and the risk of FAEs. Thus, Phase I trials were omitted due to multiple dose level and limited sample sizes. Clinical trials that met the following criteria were included in the meta-analysis: (1) prospective randomized controlled II and III trials of patients with cancer; (2) participants assigned to treatment with approved EGFR-MoAbs cetuximab and panitumumab (alone or in combination); and (3) available data on FAEs. 

### Data extraction and clinical endpoints

Data extraction was conducted independently by two investigators (L.X. and W.Z.Y.), and any discrepancy between the reviewers was resolved by consensus. For each study, the following information was extracted: author’s name, year of publication, trial phase, number of enrolled subjects, treatment arms, number of patients in treatment and control groups when available, underlying malignancy, median age, median treatment duration, median progression-free survival, adverse outcomes of interest (fatal adverse events), name and dosage of EGFR-MoAbs and the dosing schedules used. The primary end point of the analysis was treatment emergent, non-disease-related, fatal adverse events. Adverse events were defined as per versions two or three of the National Cancer Institute’s Common Terminology Criteria for Adverse Events (CTCAE) criteria[19]. Both versions are similar in defining fatal adverse events as grade five, though version three requires attribution to specific adverse events while version two did not have such requirements. We excluded events that were reported as related to disease progression, but included all events with unspecified attribution and included events regardless of attribution to treatment provided that they were not related to disease progression. 

#### Statistical analysis

We extracted the number of patients experiencing a FAEs and the total number of patients being treated with the study drug to calculate incidence. For studies with a control group in the same trial, we also calculated and compared the odds ratio (OR) of FAEs. We used the Peto method to calculate ORs and 95% confidence intervals (CIs) because this method provides the best CI coverage and is more powerful and relatively less biased than the fixed or random-effects analysis when dealing with low event rates[20]. To assess the stability of results, sensitivity analysis was carried out by sequential omission of individual studies. Additionally, to test whether effect sizes were moderated by differences in length of treatment, we have carried out meta-regressions with difference in median length of experimental treatments (expressed in weeks) as predictor and odds ratio as dependent variable. Between-study heterogeneity was estimated using the χ^2^-based Q test and I^2^ statistic[21]. The random-effects model was used in presence of heterogeneity, and the fixed-effects model in its absence. We also conducted the following prespecified subgroup analyses: different EGFR-MoAbs, phase of trials and tumor types. In addition, we calculated ORs for each study using an empirical continuity correction in all studies with zeros to investigate the sensitivity and stability of our results. We added kc=R/R+Ω to each control group cell and kt=Ω/R+Ω to the treatment arm cells. ‘R’ was calculated as the group ratio imbalance and Ω as the estimated pooled odds ratio (Mantel–Haenszel method) in the studies without zero events in both arms[20]. The presence of publication bias was evaluated by using the Begg and Egger tests [22,23]. All statistical analyses were performed by using Stata version 12.0 software (Stata Corporation, College Station, Texas, USA) and Open Meta-Analyst software version 4.16.12 (Tufts University). 

## Results

**Figure 1:**
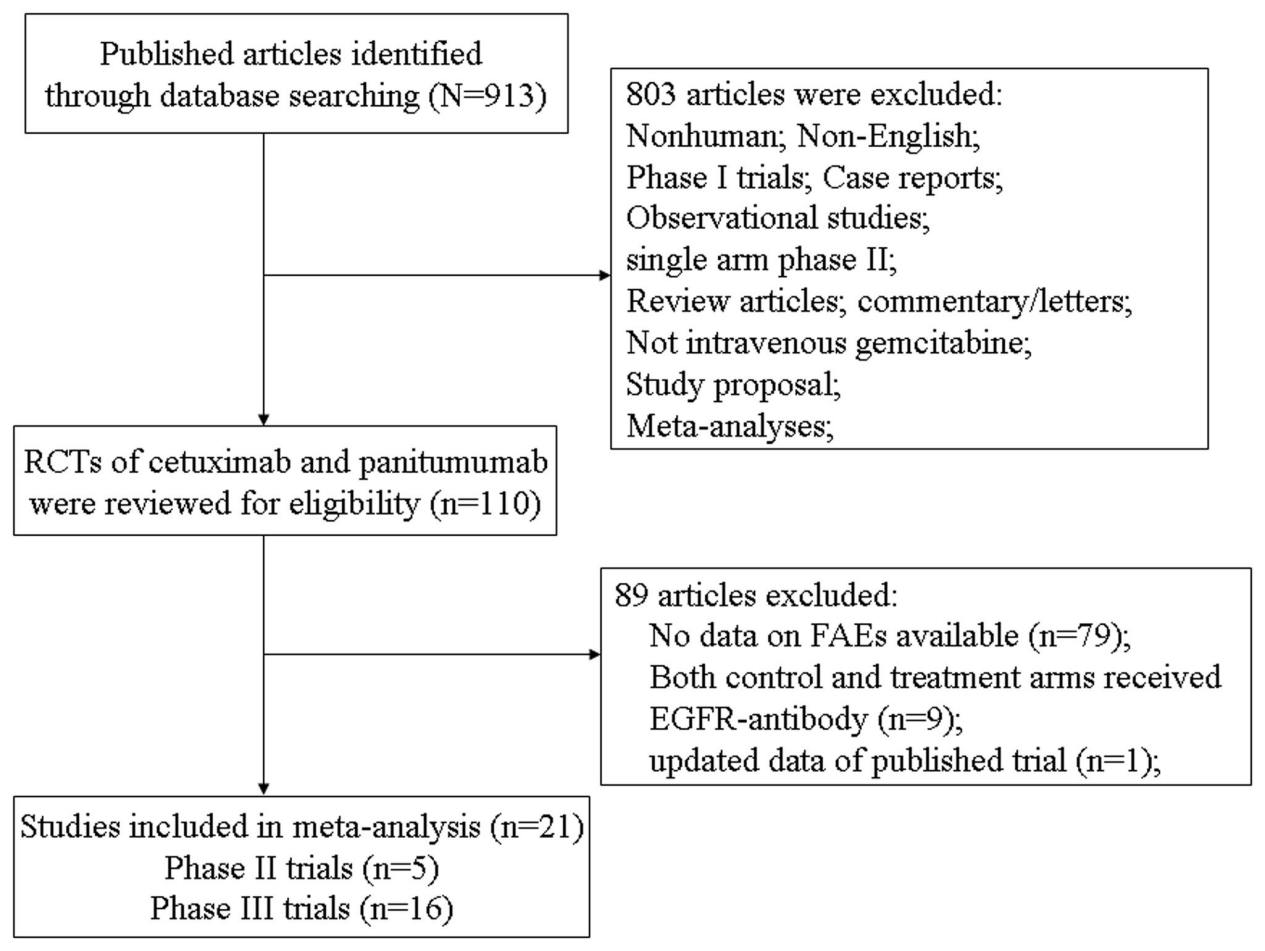
Flow chart of trial selection process in the meta-analysis.

Our search yielded 913 publications describing the use of cetuximab and panitumumab, and 21 RCTs were finally included in the meta-analysis. The selection process is summarized in Figure 1. In total, 14,776 patients were investigated in these trials and they had a variety of cancers: colorectal cancer (eleven trials)[24-34], non-small-cell lung cancer (five trials)[35-39], head and neck cancer (two trials)[40,41], esophagus carcinoma (one trial)[42], pancreatic cancer (one trial)[43] and breast cancer (one trial)[44]. Sample size were in the range of 62 to 2686 patients, with fourteen trials including >400 patients each. According to the inclusion criteria of each trial, patients were required to have an adequate renal, hepatic and hematologic function. The median age of study participants was in the range of 52–66 years (some studies only reported the mean age). Table 1 reports the study and patient characteristics for the included trials. 

**Table 1:** Baseline characteristics of the 21 trials included in the meta-analysis (*n*=14,776).

**Histology**	**Authors/ year/phase**	**Patients enrolled**	**Treatment Arm**	**Median age (years)**	**Median treatment duration (weeks)**	**Median PFS/TTP (months)**	**Median OS (months)**	**No. for analysis**	**No. of FAEs**
**CRC**	Van Cutsem E. et al/2007/III	463	P+BSC	62	NR	8weeks	NR	229	0
			BSC	63	NR	7.3 weeks	NR	234	0
	Sobreo A.F. et al/2008/III	1298	C+CPT-11	61	14	4	10.7	638	5
			CPT-11	62	13.1	2.6	10	629	2
	Bokemeyer C. et al/2009/III	341	C+FOLFOX-4	62	24	7.2	NR	170	9
			FOLFOX-4	60	NR	7.2	NR	168	5
	Hecht J.R. et al/2009/IIIB	1053	P +L-OHP+Bev	61	NR	10	19.4	407	5
			L-OHP+Bev	62	NR	11.4	24.5	397	0
			P+Bev+CPT-11	60	NR	10.1	20.7	111	2
			Bev+CPT-11	59	NR	11.7	20.5	113	0
	Van Cutsem E. et al/2009/III	1217	C+ FOLFIRI	61	25	8.9	19.9	600	25
			FOLFIRI	61	26	8	18.6	602	24
	Tol J et al/2009/III	755	C+ capecitabine +L-OHP+Bev	62	24	9.4	19.4	366	10
			Capecitabine +L-OHP+Bev	62	28	10.8	20.3	366	7
	Maughan T.S. et al/2011/III	1630	C +chemotherapy	63	NR	8.6	17	815	9
			chemotherapy	63	NR	8.6	17.9	815	10
	Alberts S.R. et al/2012/III	2686	C+ mFOLFOX6	NR	NR	NR	NR	1349	8
			mFOLFOX6	NR	NR	NR	NR	1337	3
	Saltz L. et al/2012/III	247	C +Bev+FOLFOX	63.2	24	8.3	19.5	123	6
			Bev+FOLFOX	61.2	NR	11	21.3	124	4
	Douillard J.Y. et al/2010/III	1183	P+FOLFOX4	NR	NR	9.6	23.9	539	6
			FOLFOX4	NR	NR	8.6	19.7	545	5
	Peeters M. et al/2010/III	1186	P+FOLFIRI	NR	NR	5.9	14.5	539	2
			FOLFIRI	NR	NR	3.9	12.5	540	4
**NSCLC**	Butts C.A. et al/2007/II	131	C+ GEM+platinum	66	15.1	5.09	11.99	65	0
			GEM+platinum	64	13.0	4.21	9.26	66	0
	Lienbaum R. et al/2009/II/	64	C+PTX	NR	16	3.4	NR	30	1
			bortezomib+PTX	NR	8	1.9	NR	29	1
	Pirker R.et al/2009/III	1125	C+ chemotherapy	59	18	NR	11.3	548	15
			chemotherapy	60	14	NR	10.1	562	10
	Lynch T.J. et al/2010/III	676	C +chemotherapy	64	13	4.4	9.69	325	0
			chemotherapy	65	12	4.24	8.38	320	2
	Govindan R. et al/20110/II	101	C+ chemoradiotherapy	66	NR	NR	NR	53	3
			chemoradiotherapy	65	NR	NR	NR	50	2
**head and neck cancer**	Bonner J.A.et al/2006/III	424	C+ radiotherapy	56	8	24.4	49	208	0
			radiotherapy	58	NR	14.9	29.3	212	0
	vermorken J.B. et al/2008/III	442	C+ chemotherapy	56	18	5.6	10.1	219	10
			chemotherapy	57	15	3.3	7.4	215	7
**esophagus carcinoma**	Lorenzen S. et al/2009/II	62	C+5-FU+DDP	61	16	5.9	9.5	32	0
			5-FU+DDP	62	12	3.6	5.5	30	1
**pancreatic cancer**	Philip A.P. et al/2010/III	745	C +GEM	64.3	NR	3.4	6.3	361	3
			GEM	63.7	NR	3	5.9	355	0
**MBC**	Baselga J. et al/2013/II	181	C +DDP	53	13.6	3.7	12.9	114	0
			DDP	52	13.1	1.5	9.4	57	0

Abbreviations: PFS, progression-free survival; OS, overall survival; CRC, colorectal cancer; NSCLC, non-small-cell lung carcinoma; MBC, metastatic breast cancer; FAEs: fatal adverse events; C, cetuximab; P, panitumumab; GEM, gemcitabine; BSC, best support care; L-OHP, oxaliplatin; CPT-11, irinotecan; DDP, cisplatin; Bev, bevacizumab; 5-FU, 5-fluorouracil; NR, not reported.

### Incidence of FAEs

**Figure 2:**
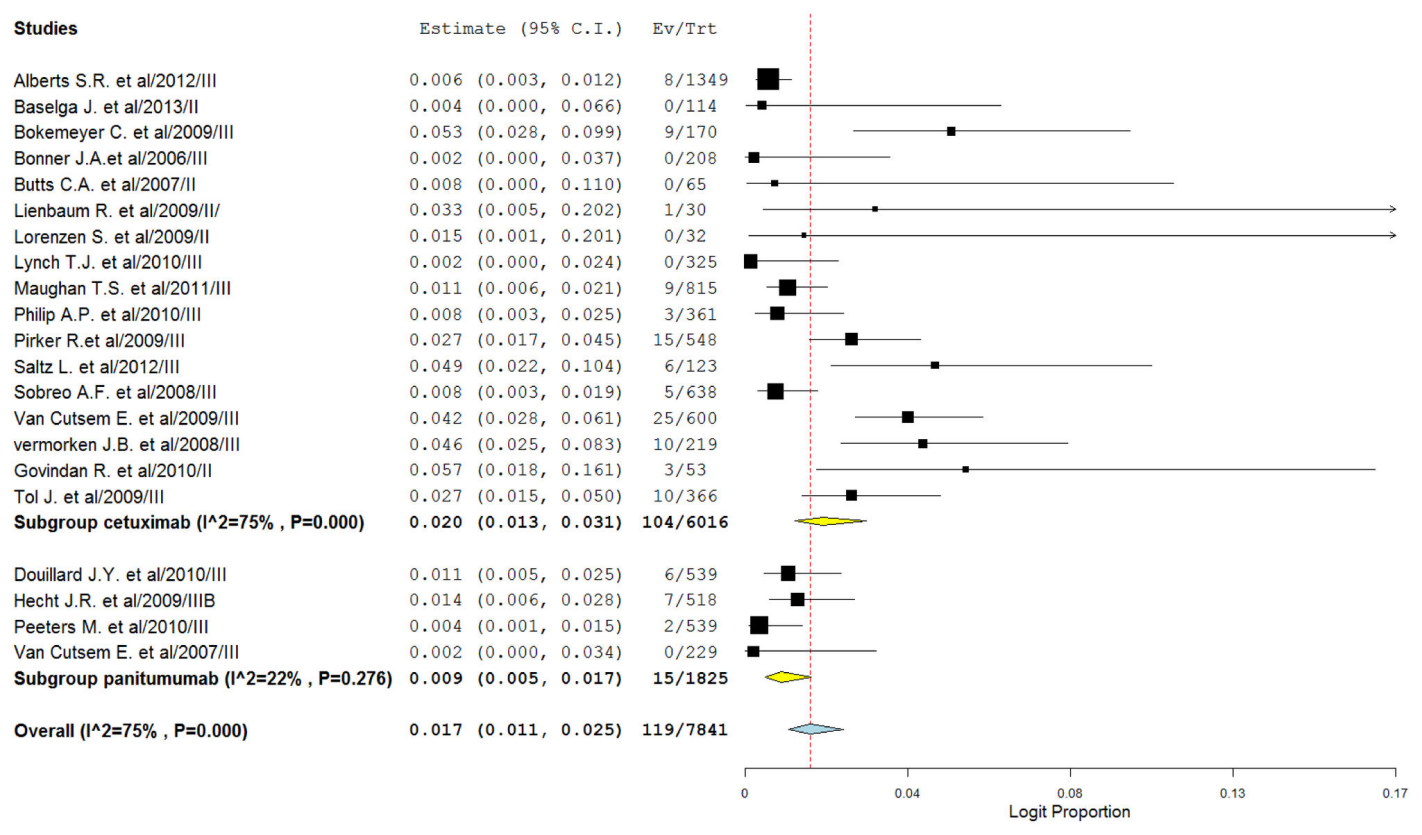
Forest plot for meta-analysis of incidence of FAEs in cancer patients assigned EGFR-MoAbs.

For the incidence of FAEs, all MoAbs treatment arms were included, representing a total of 7841 patients. By using a random-effects model (heterogeneity test: *I* ^2^=75%; *p*<0.001), the incidence of FAEs due to MoAbs was determined to be 1.7% (95%CI: 1.1-2.5%). The highest incidence was observed in a phase II trial of cetuximab in non-small-cell lung cancer [39], with a combined incidence of 5.7% (95%CI: 1.8-16.1%). No FAEs were observed in six trials[24,35,38,40,42,44], When stratified by each MoAbs, the incidence was 2.0% (95%CI: 1.3-3.2%) for cetuximab, 0.9% (95%CI: 0.5-1.7%) for panitumumab (Figure 2). The incidence of FAEs associated with cetuximab was higher than that of panitumumab (2.0% versus 0.9%, *p*=0.007). However, it should be noted that some studies with unusually broad confidence intervals of cetuximab related FAEs were also included for analysis [35,36,39,42], which might indicate less precise results. After excluding there trials, similar incidence of cetuximab related FAEs were observed (1.9%, 95%CI: 1.1-3.0%).

### Odds ratio of FAEs

**Figure 3:**
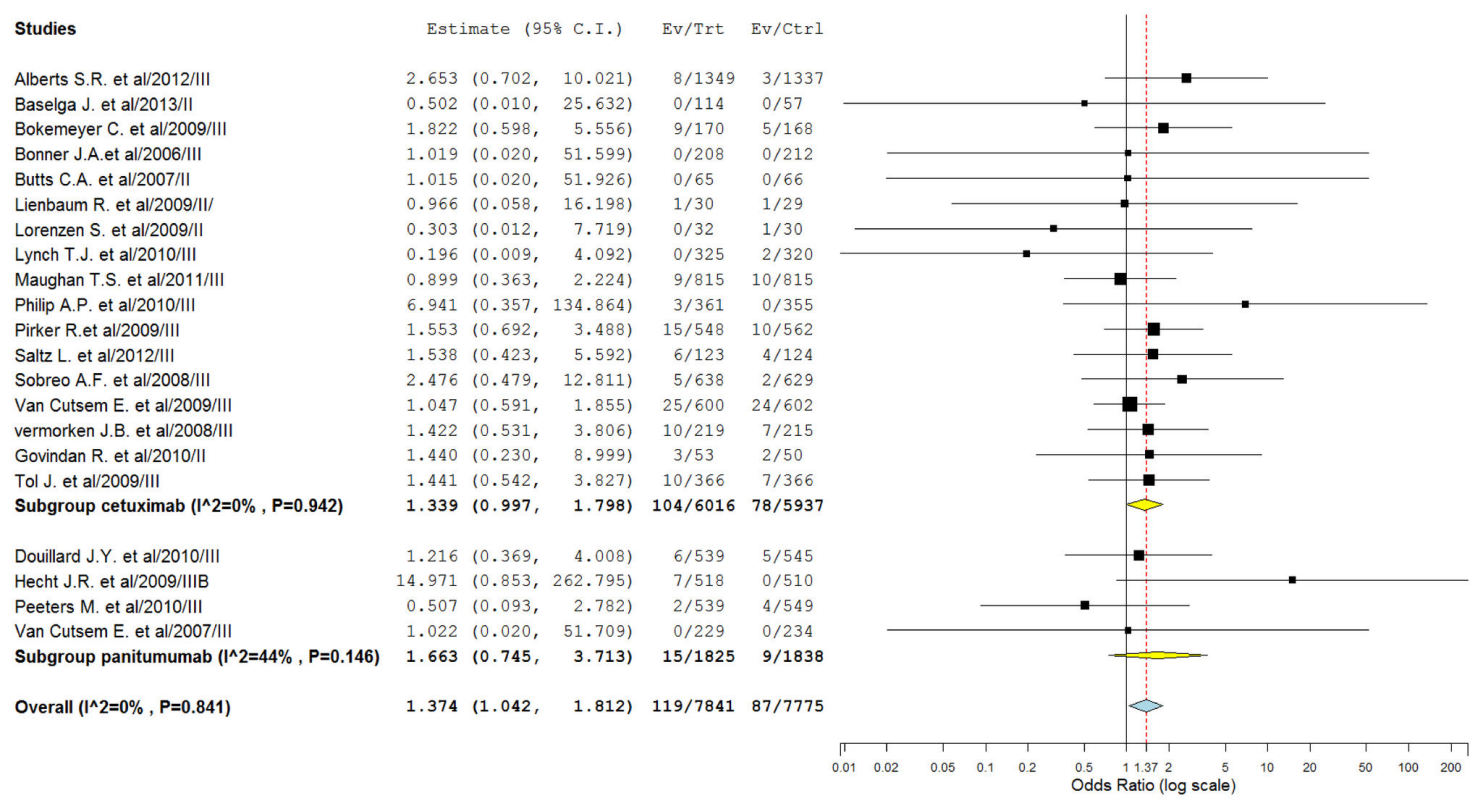
Odds ratio of EGFR-MoAbs associated FAEs versus control from randomized controlled trials of patients with cancer.

**Figure 4:**
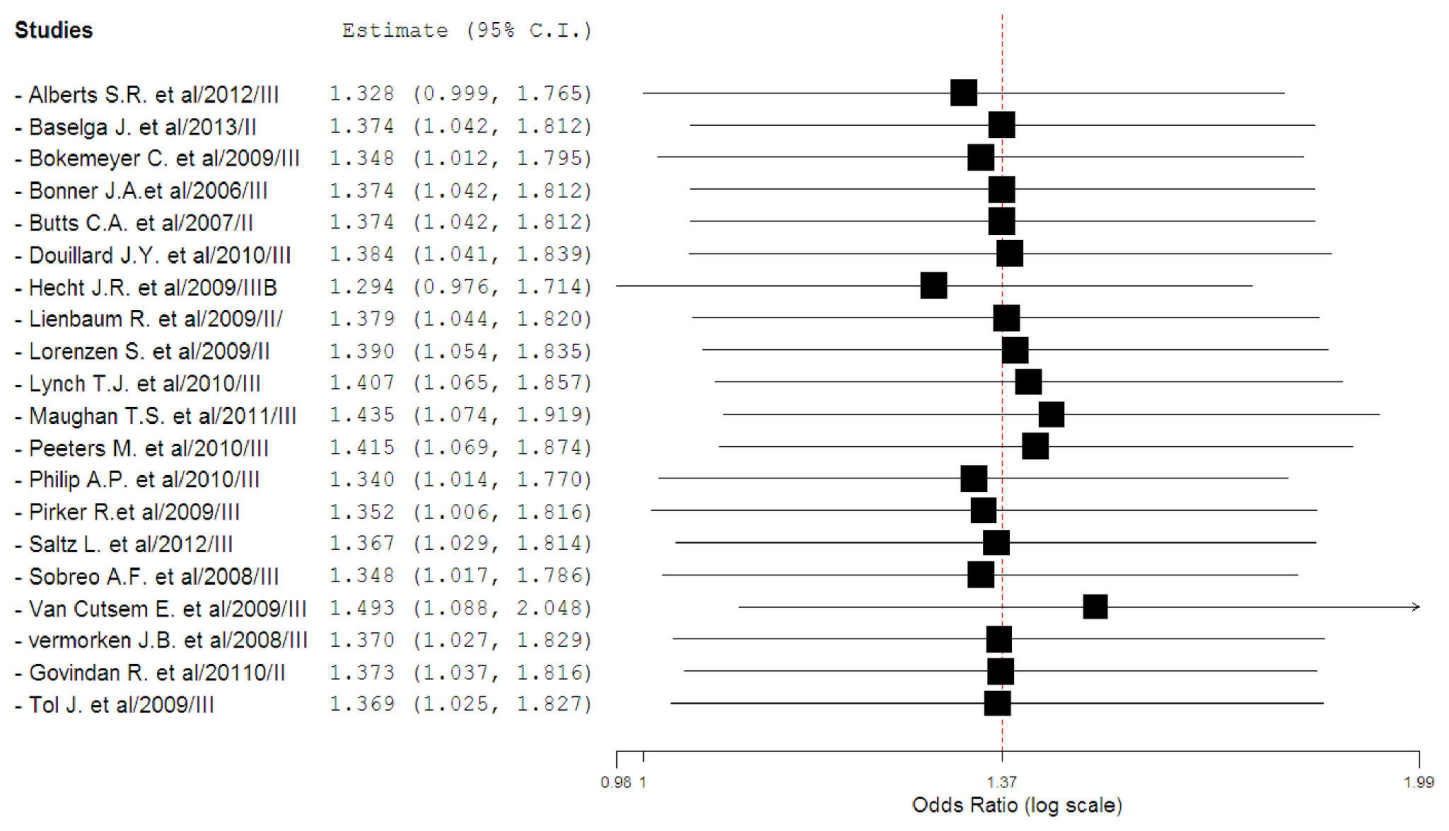
Meta-analysis of FAEs associated with EGFR-MoAbs versus control: ‘leave-one-out’ sensitivity analysis.

To investigate the specific contribution of MoAbs to the development of FAEs and exclude the influence of confounding factors such as underlying malignancy, and other therapeutic interventions, we therefore determined the odds ratio (OR) of MoAbs associated FAEs. The pooled results demonstrated that the use of MoAbs significantly increase the risk of developing FAEs with (Peto OR 1.37, 95%CI: 1.04-1.81, *p*=0.024, Figure 3) using a fixed-effect model (*I* ^2^=0%, *p*=0.84). As some included trials had a wide variation in confidence intervals, which might decrease the precision of combined results, we thus did sensitivity analysis to examine the stability and reliability of the pooled ORs by sequential omission of individual studies. The results indicated that the significance estimate of pooled ORs was not significantly influenced by omitting any other single study except for the trial conducted by Hecht J.R.[27] (Figure 4). We then performed a meta-regression analysis to test whether the OR of FAEs varied as function of difference in treatment duration of MoAbs. Since in 8 studies data about treatment duration of MoAbs were not reported, 13 of 21 studies were included in the analysis. Result indicated that there was no significant association between odds ratio and treatment duration of MoAbs (β=0.25; *p*=0.798). Additionally, sub-group analysis found that the use of cetuximab (OR 1.34, 95%CI: 1.00-1.80, *p*=0.052) and panitumumab (OR 1.66, 95%CI: 0.75-3.71; *p*=0.214) was associated with a non-significant increased risk of FAEs. Then, we explored the risk of FAEs among tumor types, and a non-significantly increased risk of FAEs is observed in CRC, NSCLC, head and neck cancer, and pancreatic cancer, while the risk of FAEs did not increase in metastatic breast cancer and esophagus carcinoma. In addition, the highest OR of FAEs was observed in pancreatic cancer patients with OR of 6.94 (95%CI, 0.36-134.87, *p*=0.731). Of note, the confidence intervals were unusually broad indicating low precision. Interestingly, there was a significantly increased risk of FAEs in phase III trials (OR 1.40, 95%CI: 1.05-1.85, *p*=0.021), but the risk did not increase in phase II trials (OR 0.95, 95%CI: 0.23-3.87, *p*=0.939) (table 2). 

**Table 2:** Incidence and relative risk of FAEs with EGFR-MoAbs according to tumor types, EGFR-MoAbs and phases of trials.

**Groups**	**Studies, n**	**Fatal Adverse Events, No./Total No.**		**Incidence (95%CI), %**	**OR (95% CI**)	**Statistical model**	***P* value**
		**EGFR-MoAbs**	**Control**				
**Overall**	21	119/7841	87/7775	1.7 ( 1.1-2.5)	1.37 (1.04-1.81)	Fixed	0.024
**Tumor types**							
CRC	11	87/5886	64/5879	1.6 (0.9-2.7)	1.37 (0.99-1.89)	Fixed	0.058
NSCLC	5	19/1021	15/1027	2.5 (1.1-5.6)	1.28(0.65-2.53)	Fixed	0.457
Head and neck cancer	2	10/427	7/427	1.4 (0.1-20.5)	1.42 (0.54-3.73)	Fixed	0.482
**EGFR-MoAbs**							
Cetuximab	17	104/6016	78/5937	2.0 (1.3-3.2)	1.34 (1.00-1.80)	Fixed	0.052
Panitumumab	4	15/1825	9/1838	0.9 (0.5-1.7)	1.66 (0.75-3.71)	Fixed	0.214
**Phases of trials**							
Phase II	5	4/294	4/232	2.8 (1.1-6.7)	0.95 (0.23-3.87)	Fixed	0.939
Phase III	16	115/7547	83/7543	1.6 (1.0-2.5)	1.40 (1.05-1.85)	Fixed	0.021

Abbreviations: NSCLC, non-small-cell lung cancer; CRC, colorectal cancer; OR, odds ratio.

### Sensitivity analysis

Sensitivity analyses using an empirical continuity correction [20] (fixed Mantel-Haenszel OR: 1.37, 95%CI: 1.04-1.80), with a continuity of 0.5 correction (fixed Mantel-Haenszel OR: 1.38, 95%CI: 1.04-1.82), or without a continuity correction (fixed Mantel-Haenszel OR: 1.35, 95%CI: 1.03-1.77) showed results similar to those of the primary analysis (table **3**). 

**Table 3:** Sensitivity analyses for the outcome of FAEs.

**Sensitivity analysis**	**Statistical model**	**OR (95%CI)**
Empirical continuity correction	Fixed (MH)	1.37 (1.04-1.80)
No continuity correction	Fixed (MH)	1.35 (1.03-1.77)
A continuity correction of 0.5	Fixed (MH)	1.38 (1.04-1.82)

Abbreviation: CI, confidence interval; MH, Mantel-Haenszel test.

### Publication bias

No evidence of publication bias was detected for the OR of FAEs in this study by either Begg or Egger’s test (OR of FAEs: Begg’s test *p*=0.343; Egger’s test *p*=0.071). 

## Discussion

This meta-analysis provides a comprehensive assessment of the risk of FAEs associated with EGFR-MoAbs. A total of 14,776 patients who received either MoAbs or non-MoAbs regimens are identified from 21 clinical trials. Our pooled results indicate that the overall incidence rate of FAEs is 1.7% (95%CI: 1.1-2.5%). Sub-group analysis demonstrates that the incidence of cetuximab-related FAEs is higher than that of panitumumab (2.0% versus 0.9%). For which we suggest two possible explanations: 1) differences in the distribution of tumor types: in our study, panitumumab is only used in colorectal cancer, while cetuximab has been used in many other tumor types including CRC, NSCLC, or head and neck cancer; 2) relative small patients receive panitumumab therapy, thus the power to detect the incidence of FAEs is low. Additionally, we also demonstrate that there is a small, but significantly increased risk of death with these agents when compared to controls, and sensitivity analysis using different statistical models also confirm our pooled results. 

We then perform subgroup analysis to explore the potential risk factors for FAEs. Our study finds that the use of cetuximab and panitumumab is associated with a non-significant increased risk of FAEs. Then, we explore the risk of FAEs among tumor types, and a non-significantly increased risk of FAEs is also observed in CRC, NSCLC, head and neck cancer, and pancreatic cancer, while the risk of FAEs does not increase in metastatic breast cancer and esophagus carcinoma. Interestingly, there is a significantly increased risk of FAEs in phase III trials (OR 1.40, 95%CI: 1.05-1.85, *p*=0.021), but the risk does not increase in phase II trials (OR 0.95, 95%CI: 0.23-3.87, *p*=0.939). There are several possible explanations for these findings: the small number of events recorded; under-reporting of rare adverse events in clinical trials (only 18% of included trials reported FAEs associated with EGFR-MoAbs); the fact that clinical trials are usually not designed specifically to address toxic events; and the small number of trials in other tumor types included. In addition, the meta-regression indicates that there is no significant association between the risk of FAEs and treatment duration of MoAbs (β=0.25; *p*=0.798). Based on our results, we could conclude that the use of EGFR-MoAbs is associated with a significantly increased risk of developing FAEs in cancer patients, and the risk of FAEs is not associated with the treatment duration of MoAbs therapies. 

Our meta-analysis has some limitations. First, this is a meta-analysis based on published data, and confounding variables at the patient level, such as co-morbidities, concomitant medications, specific age and previous therapies could not be incorporated into the analysis. Another limitation was that all of the included studies were conducted in patients with adequate organ function at study entry, suggesting that rates of FAEs could be higher in common practice. Also the process by which individual clinicians in trials determined whether a patient’s death was the result of a study drug, cancer progression or other unrelated causes carried some subjectivity and was a potential source of bias. Additionally, different treatment strategy, duration, and regimens contributed to increase the clinical heterogeneity of the meta-analysis, which made the interpretation of the meta-analysis more problematic, although we performed sub-group analysis and sensitivity analysis. And it is possible that the concomitant administration of other drugs (such as the combination of cetuximab and bevacizumab) in a few of the trials may have contributed to a higher risk of FAEs.

In conclusion, the use of EGFR-MoAbs therapies is associated with a small, but significant increase in the risk of fatal drug-related events. Despite these findings, both cetuximab and panitumumab benefit the overall population of patients with clear FDA-approved indications. As this class of drugs gains greater clinical use, clinicians should be aware of the risks associated with their use and should monitor closely and appropriately use strategies to improve patient outcomes. 

## Supporting Information

Checklist S1 (DOC)
